# Structural insights into YfiR sequestering by YfiB in *Pseudomonas aeruginosa* PAO1

**DOI:** 10.1038/srep16915

**Published:** 2015-11-23

**Authors:** Shanshan Li, Tingting Li, Yueyang Xu, Qionglin Zhang, Wei Zhang, Shiyou Che, Ruihua Liu, Yingying Wang, Mark Bartlam

**Affiliations:** 1 State Key Laboratory of Medicinal Chemical Biology, Nankai University, Tianjin, China; 2 College of Life Sciences, Nankai University, Tianjin, China; 3 Key Laboratory of Pollution Processes and Environmental Criteria (Ministry of Education), College of Environmental Science & Engineering, Nankai University, Tianjin, China

## Abstract

YfiBNR is a tripartite signalling system in *Pseudomonas aeruginosa* that modulates intracellular c-di-GMP levels in response to signals received in the periplasm. YfiB is an outer membrane lipoprotein and presumed sensor protein that sequesters the repressor protein YfiR. To provide insights into YfiBNR function, we have determined three-dimensional crystal structures of YfiB and YfiR from *P. aeruginosa* PAO1 alone and as a 1:1 complex. A YfiB(27–168) construct is predominantly dimeric, whereas a YfiB(59–168) is monomeric, indicating that YfiB can dimerize via its N-terminal region. YfiR forms a stable complex with YfiB(59–168), while the YfiR binding interface is obstructed by the N-terminal region in YfiB(27–168). The YfiB-YfiR complex reveals a conserved interaction surface on YfiR that overlaps with residues predicted to interact with the periplasmic PAS domain of YfiN. Comparison of native and YfiR-bound structures of YfiB suggests unwinding of the N-terminal linker region for attachment to the outer membrane. A model is thus proposed for YfiR sequestration at the outer membrane by YfiB. Our work provides the first detailed insights into the interaction between YfiB and YfiR at the molecular level and is a valuable starting point for further functional and mechanistic studies of the YfiBNR signalling system.

*Pseudomonas aeruginosa* is a versatile opportunistic pathogen that gives rise to numerous acute and chronic infections in humans, posing a particularly high risk for cystic fibrosis^1^ and immunocompromised individuals^2^. *P. aeruginosa* has acquired a high level of drug tolerance due to the membrane-permeability barrier^3^, which poses significant challenges for treating patients infected with this pathogen and presents a need for more efficient treatments against *P. aeruginosa* infection. Critical traits that contribute towards the pathogenicity of *P. aeruginosa* include the production of incalculable virulence factors, formation of biofilms and antibiotic resistance^4^. Biofilm formation in *P. aeruginosa* is the crucial step in persistence in the host and establishment of chronic infections, and is also responsible for cell growth and communication^5^,^6^. *P. aeruginosa* undergoes phenotypic and genetic adaptation to the lung environment during long term chronic lung infections in cystic fibrosis, including formation of mucoid cells^7^ and small colony variants (SCVs)^8^,^9^. The latter are slow-growing isolates exhibiting strong surface attachment, auto-aggregation, and enhanced exopolysaccharide production and biofilm formation. For the above reasons, *P. aeruginosa* is widely used as a model organism in the study of biofilm formation^10^.

The messenger cyclic di-GMP (c-di-GMP) triggers a number of cellular responses relevant to pathogenesis in *P. aeruginosa* and other bacteria, including motility, secretion, cytotoxicity, and biofilm formation^11^,^12^. Biofilms, which are a sessile community life form, are implicated in chronic infections and induce tolerance or resistance to host defence and antibiotic treatment, largely as a result of the extracellular matrix that holds the cells together and protects the bacterial inhabitants^13^. Enhanced biofilm formation is attributed to high cellular c-di-GMP concentration, whereas low c-di-GMP levels lead to an impairment of biofilm formation and cytotoxicity^14^,^15^,^16^. Understanding how the c-di-GMP levels are regulated is therefore vital for developing new and effective therapies against chronic infection caused by bacterial pathogens. SCVs in particular, which are linked to increased c-di-GMP levels, are correlated with prolonged persistence of infection, poor lung function, and increased resistance to antibiotics and serum.

The tripartite signalling system YfiBNR in *P. aeruginosa* modulates intracellular c-di-GMP levels in response to signals received in the periplasm and is a key regulator of the SCV phenotype^17^. Homologs of YfiBNR are widespread and affect biofilm formation in *Escherichia coli*, *Klebsiella pneumonia* and *Pseudomonas fluorescens* SBW25 through increased swimming motility^18^, cellulose production^19^,^20^ or Type 3 fimbriae expression^21^. The effector protein YfiN (also known as TpbB or PA1120) is an integral inner membrane protein that functions as a diguanylate cyclase, consisting of a periplasmic PAS domain, a cytoplasmic HAMP domain and a catalytic GGDEF domain. The periplasmic protein TpbA can dephosphorylate the PAS domain of YfiN to negatively regulate biofilm formation and negatively control c-di-GMP concentrations^22^, while the HAMP domain is required for dimerization and catalysis by the GGDEF domain^23^. YfiB, which possesses an OmpA-like peptidoglycan-binding domain, is presumed to be a sensor protein and is located in the outer membrane^17^,^24^. Mechanistic understanding of YfiB is limited, but it is believed to transduce envelope stress into rapid c-di-GMP increase inside the cell and biofilm formation via activation of the Pel and Psl exopolysaccharide systems^24^. The third component of the YfiBNR signalling system, YfiR, has a key role in the signal transduction process by bridging YfiN in the inner membrane and YfiB in the outer membrane. YfiR interacts with the periplasmic PAS domain of YfiN and is thought to function by allosterically inhibiting YfiN activity. YfiB can release the repression of YfiN by sequestering YfiR at the outer membrane, which requires both membrane anchoring and peptidoglycan binding by YfiB for full activity^24^. While little is known about YfiR function, deletion of *yfiR* causes a motility defect in *E. coli* and increased production of cellulose in both *E. coli* and *K. pneumoniae*^25^,^26^, and YfiR can be oxidized by DsbA to form intraprotein disulfide bonds that stabilize YfiR in the periplasm^26^,^27^.

Structural and mechanistic information for the YfiBNR tripartite signalling system is currently limited. To provide insights into the structure and function of this system, we have determined high-resolution crystal structures of YfiB, YfiR and the YfiB-YfiR complex from *Pseudomonas aeruginosa* PAO1. Structures of dimeric YfiB(27–168) and monomeric YfiB(59–168) reveal that YfiB can form a dimer via its N-terminal region, as confirmed by analytical ultracentrifugation. YfiR crystallizes as a dimer and features two pairs of conserved cysteine residues; Cys71-Cys110 are in a reduced state while Cys145-Cys152 form a disulfide bond. A crystal structure of YfiR with YfiB(59–168) confirms that they form a stable 1:1 complex . The YfiB-binding interface on YfiR is highly conserved and includes residues that have previously been shown to interact with YfiN. Comparison of YfiB structures in the free and YfiR-bound states reveals unwinding of the N-terminal residues from 29 to 59 reminiscent of the motility protein MotB, suggesting that this N-terminal region of YfiB reconfigures to serve as a linker for attachment of the OmpA-like domain to the outer membrane. This is consistent with the isolation of activating *yfiB* alleles with increased surface attachment and biofilm formation, all of which feature one or more substitutions in the region from residues 35 to 55. Taken together, our crystal structures enable us to propose a model for the sequestration of YfiR at the outer membrane by YfiB. This work provides the first detailed insights into the interaction between YfiB and YfiR at the molecular level and should provide a valuable starting point for further functional and mechanistic studies of the YfiBNR signalling system.

## Results & Discussion

### Crystal structures of YfiB(27–168) and YfiB(59–168)

**Figure 1:**
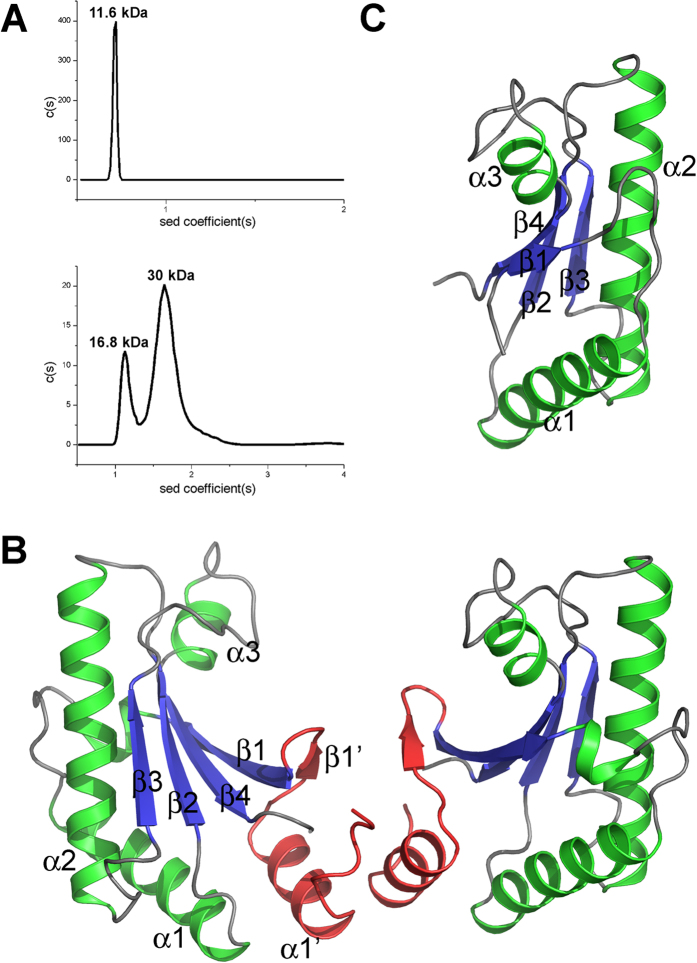
Crystal structures of *P. aeruginosa* YfiB. (**A**) Analytical ultracentrifugation data for YfiB(59–168) (top) and YfiB(27–168) (bottom). (**B**) Crystal structure of the dimeric YfiB(27–168). The structure is coloured according to secondary structure (β-strand, blue; α-helix, green), and the N-terminal residues that form the dimerization interface are coloured red. (**C**) Crystal structure of the monomeric YfiB(59–168), shown in the same orientation and colour scheme as YfiB(27–168) in panel B.

Crystal structures of the periplasmic domain of YfiB were determined for two different constructs: from residues 27–168 to 1.58 Å resolution, and from residues 59–168 to 1.39 Å resolution (Table 1). An N-terminal signal peptide from residues 1–25 and the lipid acceptor residue Cys26 were excluded from structural analysis. The YfiB(59–168) construct also excludes the region from residues 35–55 linked to increased membrane attachment. The longer YfiB construct crystallizes with two molecules in an asymmetric unit while the shorter construct crystallizes with only one molecule per asymmetric unit. Analytical ultracentrifugation data confirms that YfiB(27–168) predominantly forms a dimer in solution, while YfiB(59–168) is exclusively a monomer (Fig. 1A).

The structure of YfiB(27–168) is an OmpA-like domain with a core domain consisting of helices α1–3 and a four-stranded anti-parallel β-sheet with topology β1-β4-β2-β3 (following the numbering for the core conserved domain of Pal^28^). The N-terminal fragment visible from residues 30–53 includes an additional strand β1’ that packs anti-parallel to β1 in the core β-sheet, and helix α1’ that lies parallel to helix α2 (Fig. 1B). The YfiB(59–168) structure lacks helix α1’ and strand β1’ and has a considerably shorter β1 strand than its larger counterpart, but otherwise retains the same core domain (Fig. 1C). The two structures can be superimposed with an r.m.s.d. of 1.3 Å for 105 aligned residues. The largest structural differences occur at the N-terminus, which dissociates from the β-sheet in YfiB(59–168), and in the β2-α2 loop, which packs against helix α3 in the shorter structure but is directed away from α3 in the longer structure. A Dali search reveals closest structural homology to the inner membrane protein Yiad (PDB ID: 2K1S, Z score 18.0, r.m.s.d. 2.3 Å for 134 aligned residues, 32% sequence identity, unpublished), motility protein MotB (PDB ID: 3S06, Z score 18.0, r.m.s.d. 1.8 Å for 120 aligned residues, 25% sequence identity), the C-terminal periplasmic region of PomB (PDB ID: 3WPW, Z score 17.9, r.m.s.d. 2.0 Å for 130 aligned residues, 28% sequence identity), and OmpA (PDB ID: 4RHA, Z score 15.7, r.m.s.d. 1.8 Å for 113 aligned residues, 36% sequence identity).

The YfiB(27–168) structure reveals a weak asymmetric dimer whose interface buries approximately 421 Å^2^, or about 5.5% of the total surface area for each monomer, with a solvation free energy gain ΔG of −4.7 kcal mol^−1^ upon formation of the interface as calculated with PISA^29^ (Fig. 1B). The predominantly hydrophobic dimer interface is formed by the N-terminal helix α1’ and strand β1’ of each monomer. The interfacing residues are mainly on helix α1’ and include Leu35, Ser36, Ala37, Glu38, Ile40, Ala41 and Gln44, with additional contributions from Phe48, Glu49 and Leu50 on strand β1’ and Trp55 on strand β1 (Supplementary Figure S1). Gln44 in subunit A contributes hydrogen bonds with the main chain atoms of Ala37 and Leu35 in subunit B, while Gln44 in subunit B contributes one hydrogen bond with Gln32 in subunit A. Mutation of certain residues in this region can alter the propensity of YfiB to form a dimer: L43P is predominantly a monomer by AUC, F48S exists as a mixture of monomer and dimer, and W55L is predominantly a dimer (Supplementary Figure S2). Leu43 is not involved in dimerization, but it is in close proximity to the dimer interface and the L43P mutant is reported to have increased membrane attachment^24^.

Our structures confirm previous predictions that YfiB has a conserved OmpA-like peptidoglycan (PG) binding domain^17^,^28^. Residues implicated in PG binding by YfiB have previously been mapped by Malone and colleagues and include two critical residues, Asp102 and Gly105^24^. Asp102 is strictly conserved in *E. coli* and *K. pneumoniae* YfiB, whereas Gly105 is conserved in *K. pneumoniae* but substituted by valine in *E. coli* (Supplementary Figure S3). A gene in which these two sites were mutated had no effect on attachment and did not induce SCV morphology. Asp102 and Gly105 are located on the β2-α2 loop which, together with the β3-α3 loop, define a cavity that could accommodate PG. Attempts to co-crystallise YfiB with PG have so far proven unsuccessful, but further work is underway to confirm the location of this PG-binding site and to elucidate the mechanism of PG binding.

### Crystal structure of YfiR

**Figure 2:**
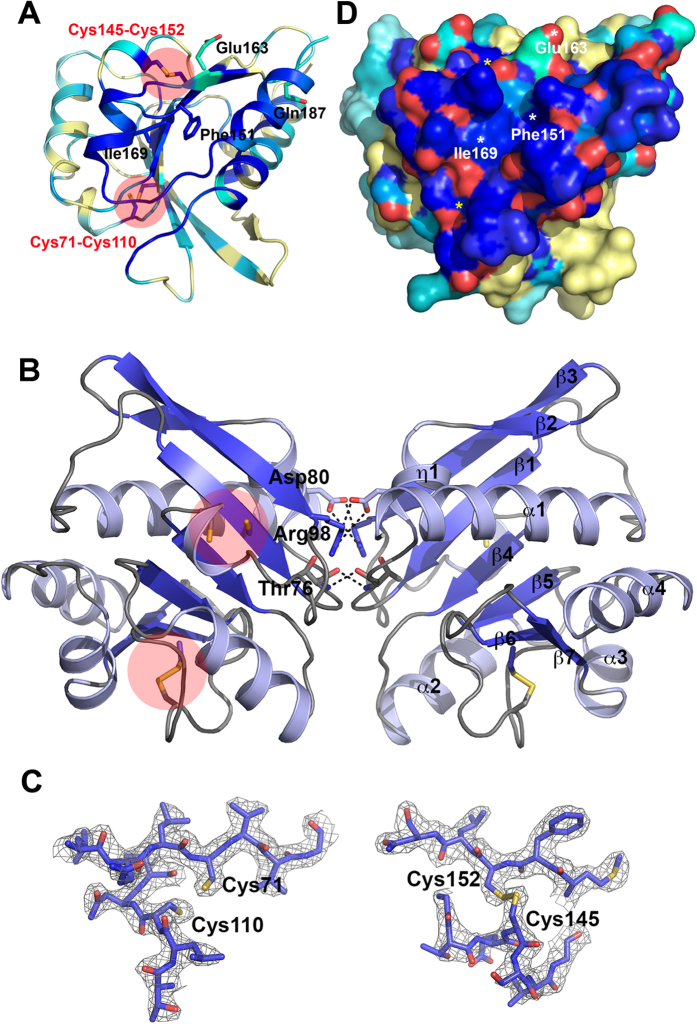
Crystal structure of *P. aeruginosa* YfiR. (**A**) Crystal structure of a YfiR monomer coloured according to sequence conservation by the ConSurf server. Pale yellow regions represent low sequence conservation and regions in blue represent high sequence conservation. Residues implicated in binding to YfiN are shown in stick representation and labelled. (**B**) The YfiR dimer structure. One monomer is coloured blue and the other monomer is coloured pale blue. (**C**) The two pairs of disulfide bonds in YfiR. Cys145-Cys152 form a disulfide bond in the crystal structure but Cys71-Cys110 is broken. Residues are shown in stick representation and are fit into 2mFo-dFC electron density (grey mesh) contoured at 2.0σ. (**D**) A surface representation of the YfiR monomer coloured according to sequence conservation (as detailed in panel A). The positions of residues implicated in binding to YfiN are labelled.

The gene encoding YfiR from residues 35–190 was amplified from the *P. aeruginosa* PAO1 genome. The N-terminal 34 amino acids encode a signal peptide and were therefore not included for structural analysis. The crystal structure of YfiR was determined to 2.40 Å resolution and reveals two molecules in an asymmetric unit, with each molecule traced in continuous electron density from Arg38 at the N-terminus to Thr189 at the C-terminus (Table 1). Each YfiR monomer consists of a twisted, seven-stranded β-sheet flanked on one side by the N- and C-terminal helices (α1 and α4 respectively), and on the other by the helices α2 and α3 (Fig. 2A). The mixed, seven-stranded β-sheet has a β2-β3-β1-β4-β5-β6-β7 topology. Analytical ultracentrifugation analysis confirms that YfiR forms a dimer in solution (Supplementary Figure S4), which is consistent with a recent YfiR crystal structure^27^. The two-fold symmetrical dimer is formed by several key interactions, including salt-bridges by Asp80 and Arg98 and main chain hydrogen bonding between Thr76 of each monomer (Fig. 2B).

YfiR is annotated as belonging to the Pfam13689 or DUF4154 family, but a Dali search reveals similarity to the ABC domain of *Streptococcus pneumonia* (PDB ID: 3LFT, Z-score: 11.4, r.m.s.d. 3.2 Å for 130 aligned residues, sequence identity: 13%, unpublished) and an uncharacterised protein from *Vibrio cholerae* (PDB ID: 3LKV, Z-score: 11.3, r.m.s.d. 3.1 Å for 131 aligned residues, sequence identity: 11%, unpublished). The *S. pneumonia* ABC domain is a type I periplasmic ligand-binding domain of an uncharacterized ABC-type transport system that is predicted to participate in the uptake of amino acids, peptides, or inorganic ions. It consists of two sub-domains, an N-terminal and C-terminal domain, which share a similar core fold and can be superimposed with an r.m.s.d. of 2.4 Å for 92 aligned residues. YfiR adopts a similar fold to the N-terminal domain of both proteins with some key differences. Most notably, strand β2 and η1 in YfiR are replaced by an α-helix in the *S. pneumonia* ABC domain, and an additional strand at the C-terminus of the *S. pneumonia* ABC domain is adjacent to the equivalent strand to β7 in YfiR^27^. The four cysteine residues present in YfiR are non-conserved in the *S. pneumonia* and *V. cholerae* protein structures.

Two highly conserved pairs of cysteine residues were identified in the structure, Cys71-Cys110 and Cys145-Cys152, with only the latter pair confirmed to form a disulfide bond from the electron density (Fig. 2C; Supplementary Figure S5). In their recent structural analysis of YfiR, Yang and colleagues observed that Cys71-Cys110 form a disulfide bond in an oxidative environment that is broken in a reducing environment^27^. Yang and colleagues further confirmed that the Cys145-Cys152 disulfide bond is essential for correct folding and stability of YfiR, which is consistent with our observations that Cys145 and Cys152 mutants exhibited reduced expression levels and poorer stability. Deletion of the gene encoding the periplasmic thiol:disulfide interchange protein DsbA, which catalyses cysteine crosslinking and regulates the correct folding of periplasmic proteins, results in a strong SCV phenotype in *P. aeruginosa* PAO1 and markedly reduced YfiR levels^24^. YfiN levels were not reduced in the same phenotype, suggesting that misfolding of YfiR leads to specific activation of YfiN. The absence of DsbA and DsbB or the presence of DTT also results in instability of YfiR in *E. coli*^26^.

Malone and colleagues used a molecular model of YfiR to propose a putative YfiN-binding surface based on the location of four C-terminal mutations^24^. As the YfiBNR signalling system is conserved across many bacterial species, we postulated that the YfiN-binding surface on YfiR should also be conserved. We therefore used the ConSurf server^30^ to estimate the evolutionary conservation of amino acids in YfiR from 149 unique homologous sequences. Mapping the conservation scores for each amino acid onto the YfiR structure reveals a large, continuous, highly conserved region on the surface of the protein formed by the C-terminal helices α3, α4 and the α3-α4 loop, with contributions from strand β7, the N-terminal helix α1 and the α1-β1 loop (Fig. 2D). Of the four mutations identified by Malone and colleagues as enhancing the interaction with YfiN, Ile169 is exposed on the surface of this conserved region and the non-conserved Glu163 is on the periphery of this region. Phe151 forms the base of a deep hydrophobic pocket on the conserved surface and might be unlikely to interact with YfiN directly. Gln187 situated on helix α4 is located on the underside of this surface, but it is conceivable that it could become repositioned upon binding to YfiN. Further work is underway to confirm whether or not this is the YfiN binding surface by structural and interaction studies.

### Crystal structure of the YfiB-YfiR complex

**Figure 3:**
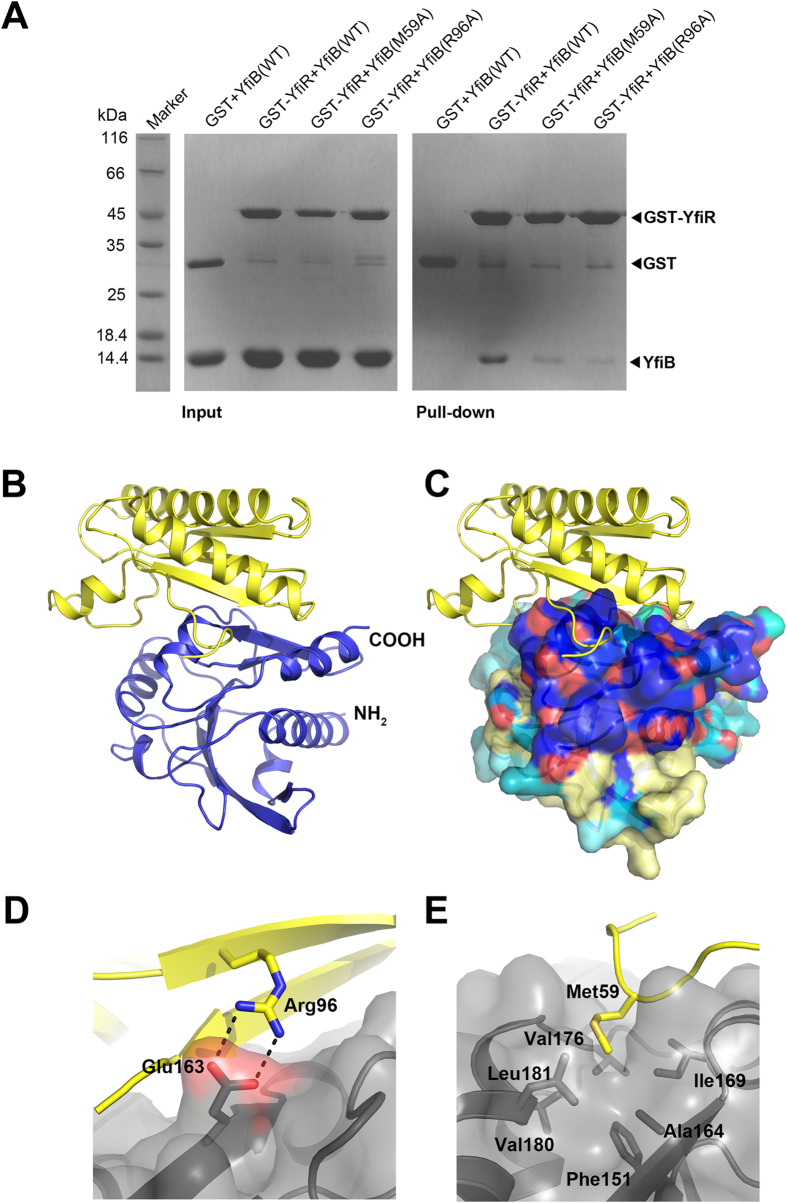
Crystal structure of the *P. aeruginosa* YfiB-YfiR(C71S) complex. (**A**) GST-pulldown of GST-YfiR with YfiB (wild-type and M59A, R96A mutants). (**B**) Crystal structure of the YfiB-YfiR complex. YfiB is shown in yellow cartoon representation and YfiR(C71S) is shown in blue. (**C**) The YfiB-YfiR complex shown in the same orientation as panel A. YfiR is shown in surface representation and coloured according to sequence conservation (as detailed in Fig. 2A). (**D**) The salt bridge formed between YfiR Gln163 (grey) and YfiB Arg96 (yellow). (**E**) Interaction of YfiB Met59 (yellow) with a hydrophobic pocket in YfiR including Phe151.

It has previously been confirmed that YfiB sequesters the repressor protein YfiR, although Malone and colleagues were unable to co-immunoprecipitate YfiB and YfiR together and it remains unclear if YfiB sequesters YfiR through direct protein-protein contact or via additional components^24^. We therefore sought to determine whether or not YfiR and YfiB can interact directly. Co-purification of YfiR and YfiB and GST pull-down indicates that the two proteins are able to form a stable complex in solution (Fig. 3A). However, only YfiB(59–168) could be crystallized in a complex with a C71S mutant of YfiR (Fig. 3); attempts to co-crystallize YfiB(27–168) with wild-type YfiR yielded poorly diffracting crystals. The resulting structure to 1.97 Å resolution reveals one YfiR monomer binds to one monomer of YfiB(59–168) (Table 1). The interface area of the complex is 1,053 Å^2^, with YfiB burying 1,024 Å^2^ (16% of the total accessible surface area) and YfiR burying 1,079 Å^2^ (13% of the total accessible surface area). The solvation free energy gain ΔG upon formation of the interface was calculated as −13.1 kcal mol^−1^ using PISA^29^.

YfiR interacts with YfiB exclusively through its C-terminal region (Fig. 3B,C). Strand β1 of YfiB is completely unwound, allowing strand β7 of YfiR to stack against strand β4 of YfiB in an anti-parallel manner to form an extended β-sheet spanning both proteins (Fig. 3B). Additional interactions are provided by helices α3 and α4 and the β4-β5 loop of YfiR. Comparison of the YfiB interface in YfiR with the conserved surface of YfiR shown in Fig. 2B shows that approximately 30% (1,030 Å^2^ of a total area of 3,478 Å^2^ calculated using the program AreaIMol in CCP4^31^) of the conserved surface overlaps with the YfiB interaction surface (Fig. 3B). Furthermore, both Glu163 and Ile169 of YfiR, which have previously been shown to interact with YfiN^24^, also interact with residues in YfiB (Fig. 3D,E), suggesting that YfiR uses this same region to bind to both YfiN in the inner membrane and YfiB in the outer membrane. Further work is required to confirm the YfiN binding interface of YfiR.

The extensive interface between YfiB and YfiR includes a number of hydrogen bonds and salt bridges: Arg96 of YfiB forms a hydrogen bond with Glu144 of YfiR and a salt bridge with Glu163 (Fig. 3C). Other hydrogen bonds are formed between Arg138 of YfiB and Ser146 of YfiR; the main chain nitrogen atom of Val165 in YfiB and the carbonyl oxygen of Ala164 in YfiR; the main chain nitrogen atom of Ser61 in YfiB and the carbonyl oxygen of Leu166 in YfiR; and between the carbonyl oxygen of Glu157 in YfiB and the NH1 atom of Arg171. Phe151, which is also reported to be important for the interaction with YfiN, does not interact directly with YfiB but is situated at the bottom of a deep hydrophobic pocket lined by Ala164, Ile169, Val176, Val180 and Leu181. Met59 of YfiB inserts into this hydrophobic pocket and interacts with the lining residues such that its side chain is 5.3 Å from Phe151 (Fig. 3D). Mutation of either Met59 or Arg96 of YfiB weakens but does not abolish the interaction with YfiR (Fig. 3A). Gln187 located on helix α4 of YfiR was also reported to be important for the interaction with YfiN; it does not interact with YfiB directly in our crystal structure, but forms a hydrogen bond with the carbonyl oxygen of Ile160 on strand β7 of YfiR and presumably helps to stabilise the binding interface. The choice of a C71S mutant of YfiR breaks the Cys71-Cys110 disulfide bond, but the Cys145-Cys152 disulfide bond remains intact in the C-terminal of the protein upon binding to YfiB. This disulfide bond appears to be critical for maintaining the local structure as surrounding residues, including Glu144 and Ser146, constitute part of the interface and form hydrogen bonds with YfiB.

**Figure 4:**
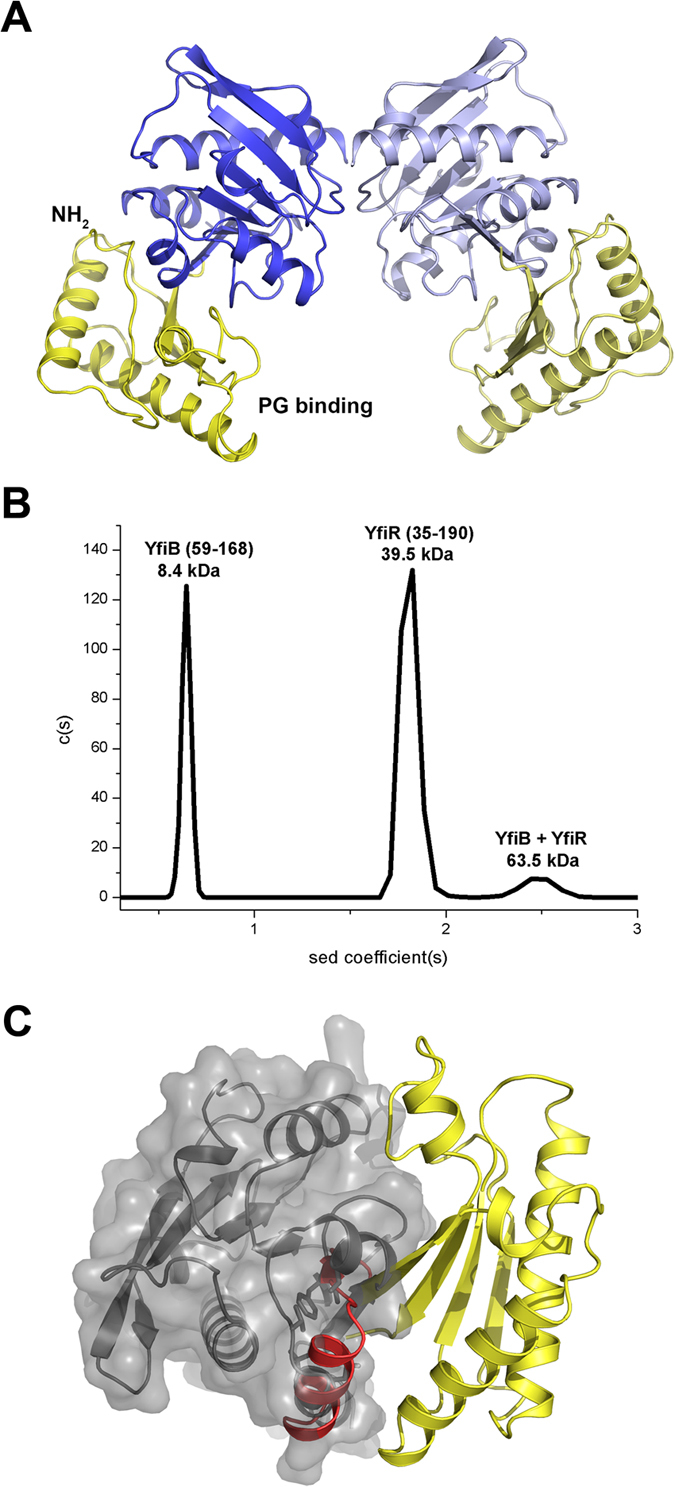
Stoichiometry of the YfiB-YfiR complex. (**A**) A YfiB-YfiR dimer observed from crystal symmetry. YfiR is coloured blue and YfiB is coloured yellow. YfiR shows an identical mode of dimerization as observed in the YfiR crystal structure. YfiR is shown in the same orientation as in Fig. 2B. (**B**) Analytical ultracentrifugation of the YfiB-YfiR complex. Peaks are observed corresponding to the YfiB monomer (8.4 kDa), the YfiR dimer (39.5 kDa) and the 2:2 YfiB-YfiR complex (63.5 kDa). (**C**) Superposition of YfiB(27–168) onto the YfiB-YfiR complex structure. The N-terminal residues shown in red clash with YfiR (shown by a transparent grey surface) in the crystal structure, indicating that this region of YfiB partly obscures the YfiR-binding interface.

YfiR-YfiB(59–168) crystallizes with one complex in an asymmetric unit. However, inspection of the crystal symmetry reveals the same mode of dimerization by YfiR as observed by us (Fig. 2C) and by Yang and colleagues for the native protein^27^. In this arrangement, YfiB binds to each end of the YfiR dimer such that the N-terminal linkers are situated at one side of the complex and the conserved PG-binding sites are located on the opposing side (Fig. 4A). In this configuration, the two YfiB monomers are unable to interact with each other. Analytical ultracentrifugation analysis of the YfiR-YfiB(59–168) complex shows a peak at 64 kDa, consistent with a 2:2 stoichiometry (Fig. 4B), as well as peaks corresponding to a YfiB(59–168) monomer and a YfiR dimer.

### A model for YfiR sequestration by YfiB

**Figure 5:**
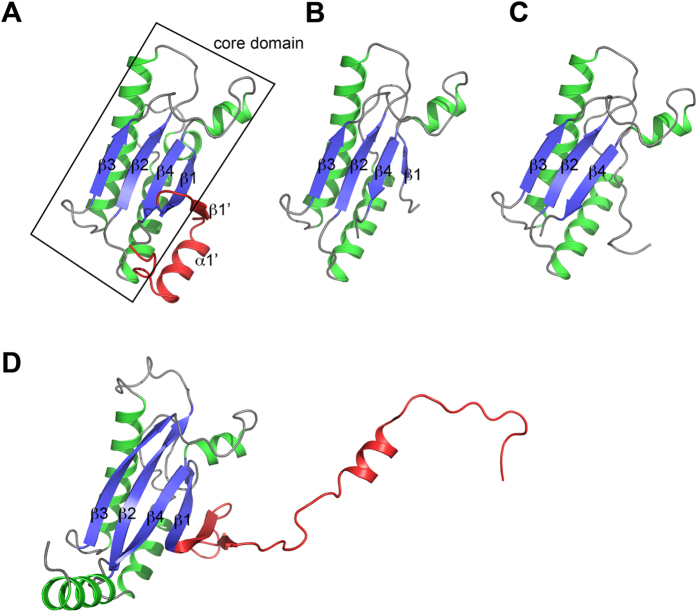
Structural changes in the N-terminal region of YfiB. Crystal structures are shown of (**A**) YfiB(27–168), (**B**) YfiB(59–168), and (**C**) YfiB(59–168) from the YfiB-YfiR complex. (**D**) Crystal structure of MotB (PDB ID: 3S0Y) for comparison. All structures are superimposed and shown in the same orientation. The core Pal- or OmpA-like domain is coloured according to secondary structure following the scheme in Fig. 1. N-terminal residues forming the putative linker are coloured red.

To investigate why YfiB(59–168) could be crystallized with YfiR but not YfiB(27–168), we superimposed our complex onto the longer YfiB construct. Following superposition, it was evident that the N-terminal region of YfiB clashes considerably with YfiR, thus inhibiting the sequestration of YfiR (Fig. 4C). This may explain why Malone and colleagues were unable to co-immunoprecipitate YfiB and YfiR together^24^. We therefore propose that YfiB would be required to undergo a conformational change in its N-terminal region in order to anchor to the outer membrane and sequester YfiR. Recent structural studies on PomB^32^ and MotB^33^, which are close structural neighbours of YfiB, indicate that a large conformational change of the PEM (Periplasmic region Essential for Motility) is required for anchoring to the PG layer. In their study on the MotB linker, O’Neill and colleagues analysed the structures of a series of N-terminally truncated MotB fragments to reveal the mechanism of linker unfolding^33^. Analogous to MotB, the core domain of YfiB consists of a four-stranded β-sheet, β1-β4, and helices α1-α3. Strand β1’ and helix α1’ includes residues that are proposed to form a linker region that unfolds to facilitate membrane attachment and YfiR sequestration. This is consistent with our observations from structures of YfiB alone and in complex with YfiR that reveal structural rearrangements in this N-terminal linker region (Fig. 5A–C), including shortening of strand β1 in YfiB(59–168) and complete unfolding of β1 upon YfiR binding. Unfolding this linker would free the N-terminal to attach to the outer membrane, similar to that proposed for MotB attachment to the inner membrane^33^ (Fig. 5D), thus exposing a number of residues in the region from 35 to 55 that have been implicated in surface attachment and biofilm formation^24^. Activating mutations in this region are dominant over the loss of PG binding and can fix YfiB in its active conformation independent of PG binding. The weak dimer observed for YfiB(27–168) may help to suppress its role in YfiR sequestration by maintaining the linker in a folded conformation and thus inhibiting YfiR binding, although further experiments are required to confirm this.

**Figure 6:**
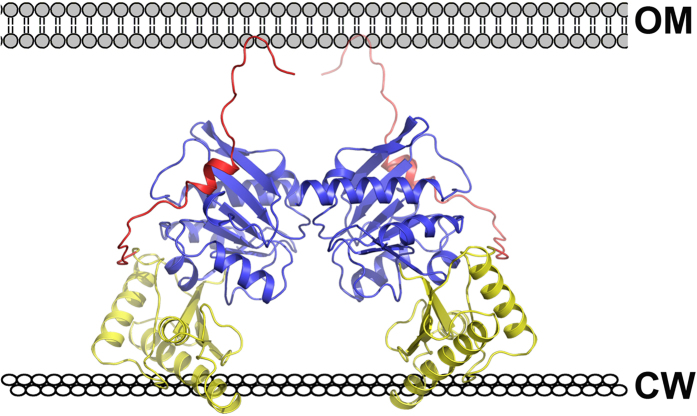
A model for sequestering of YfiR by YfiB. The YfiB-YfiR dimer is shown and coloured as in Fig. 3E. (**A**) YfiB linker shown in red is modelled from the crystal structure of MotB (PDB ID: 3S0Y) from the acceptor residue Cys26 to residue 61 in the crystal structure. The YfiB-YfiR dimer is oriented such that the PG-binding pockets interact with the cell wall (CW) and the YfiB linker extends to attach to the outer membrane (OM).

As both membrane anchoring and peptidoglycan binding by YfiB are required for activity, and mutants with strong activating effects cluster in the N-terminal region, this allows us to propose a model for YfiR sequestering by YfiB. The linker region between lipid acceptor Cys26 and the OmpA-like domain plays an important role in YfiB-mediated signalling, with a shortened linker producing stronger YfiR sequestration to the outer membrane, surface attachment and SCV morphology^24^. Malone and colleagues have suggested that YfiR sequestration by YfiB depends on the ‘wingspread’ of the linker between the outer membrane and cell wall. We constructed a model for YfiB using an appropriate length of linker from MotB as a template to approximate the region from the acceptor ligand Cys26 to residue 61 in our crystal structure. In this model, two YfiB molecules encase one YfiR dimer (Fig. 6). The N-terminal linker extends up to attach to the outer membrane, while the putative PG-binding pockets of YfiB are located at the bottom of the YfiB-YfiR complex and are suitably oriented to bind to the PG layer. The advantage of this dimeric arrangement is that YfiB can more stably and efficiently bind to the PG layer. The N-terminal linker would allow YfiB to sense changes in the distance between the outer membrane and PG layer and either expose or obscure the YfiR-binding surface, thus controlling how much YfiR is sequestered. It is not clear if YfiB directly challenges YfiN for YfiR, but this may be unlikely as YfiR uses the same region to bind both YfiB and YfiN. A more likely scenario is that YfiB removes unbound YfiR from the periplasm and shifts the equilibrium towards unbound and active YfiN^24^. A secondary role for YfiR as a cysteine-dependent redox sensor is also suggested in which YfiN is activated as a result of YfiR misfolding in a reduced environment, although the precise interplay between these two mechanisms remains to be established.

## Conclusions

Our structural analysis of components of the YfiBNR tripartite signalling system reveals that YfiR is a versatile periplasmic binding protein that interacts with YfiB via a highly conserved surface that is also predicted to interact with YfiN. To the best of our knowledge, this is the first confirmation that YfiB can interact with YfiR via direct protein-protein contact. This region of YfiR features several amino acids that were previously shown to be involved in interaction with the PAS domain of the inner membrane protein YfiN. Our series of YfiB crystal structures indicate that the N-terminal residues of YfiB obstruct the YfiR-binding interface and must unfold in order for YfiR to be sequestered at the outer membrane. This role for the N-terminal region of YfiB is consistent with its involvement in membrane attachment, as isolation of activating *yfiB* alleles with increased surface attachment and biofilm formation all include mutations in the region from residues 35–55. In summary, we have provided the first detailed insight into YfiR sequestering by YfiB at the molecular level. This work should provide a starting point for further functional and mechanistic studies of the YfiBNR tripartite signalling system, as well as for exploring the potential of targeting this network for the discovery of novel therapeutics against chronic infections caused by bacterial pathogens.

## Materials & Methods

### Plasmid construction

The two truncated genes encoding YfiB (PA1119; gene ID AAG04508.1) were amplified from the genome of *Pseudomonas aeruginosa* PAO1 (kindly provided by Prof. Lei Wang). The PCR product for the longer truncation including amino acids 27–168 was purified and digested with BamHI and XhoI. Digested PCR products encoding YfiB(27–168) were cloned into the vector pGEX-6p-1 (GE Healthcare). The PCR product for the shorter truncation including amino acids 59-168 was purified and digested with *Bam*HI and *Not*I. Digested PCR products encoding YfiB(59–168) were cloned into the vector pET-32a (GE Healthcare).

The gene encoding YfiR (PA1121; gene ID AAG04510.1; residues 35–190) was amplified from the genome of *Pseudomonas aeruginosa* PAO1 and then inserted into the expression vector pET-32 a (GE Healthcare) using the restriction sites *Bam*HI and *Not*I and with an N-terminal Trx tag.

### Protein expression and purification

For the expression and purification of YfiB(27–168), the *E. coli* strains were grown in Luria–Bertani broth medium containing 100 μg ml^−1^ ampicillin at 37 °C. When the OD_600_ of the culture reached 0.6, isopropyl β-D-1-thiogalacto-pyranoside was added to the growth medium to a final concentration of 0.3 mM to induce the expression of recombinant proteins. The induced cultures were grown at 16 °C for 16 h. Cells were harvested by centrifugation, resuspended in phosphate-buffered saline (1 × PBS pH 7.4) and then lysed by sonication on ice. The cell debris was removed by centrifugation at 18,000g for 40min at 4 °C. The supernatant was loaded on to a GST column (GE Healthcare) equilibrated with 1 × PBS pH7.4. The column was washed briefly with 1 × PBS pH 7.4 and then extensively with wash buffer consisting of 1 × PBS 1 M NaCl, pH 7.4. The GST tag was removed by adding 0.6mg PPase to the resin overnight. The protein was eluted with 1 × PBS pH7.4 then concentrated and purified using anion-exchange chromatography on a Hitrap Q column (GE Healthcare).The protein was further purified using a Superdex75 column (GE Healthcare) equilibrated with 20 mM Tris pH 8.0, 200 mM NaCl pH 8.0. The purified protein was transferred to a buffer consisting of 20 mM Tris pH 8.0, 200 mM NaCl for crystallization.

For the expression and purification of YfiB(59–168), the supernatant was loaded on to a Ni–NTA column(GE Healthcare) equilibrated with 1 × PBS pH7.4. The column was washed briefly with 1 × PBS pH7.4 and then extensively with wash buffer(1 × PBS, 20 mM imidazole). We added 0.6 mg PPase to the resin to remove the 6*His tag overnight. The protein was eluted with 1 × PBS pH 7.4 then concentrated and purified using cation-exchange chromatography on a Hitrap S column(GE Healthcare).The protein was further purified using a Superdex75 column (GE Healthcare) equilibrated with 20 mM Tris pH 8.0, 200 mM NaCl pH 8.0. The purified protein was transferred to a buffer consisting of 20mM Tris pH 8.0, 200 mM NaCl for crystallization.

For the expression of YfiR, the *Escherichia coli* strains were grown in Luria–Bertani broth medium containing 100 μg ml^−1^ ampicillin at 37 °C. When the OD_600_ of the culture reached 0.6, isopropyl β-D-1-thiogalacto-pyranoside was added to the growth medium to a final concentration of 0.3 mM to induce the expression of recombinant proteins. The induced cultures were grown at 16 °C for 16 h. Cells were harvested by centrifugation, resuspended in 1 × PBS, pH 7.0 and then lysed by sonication on ice. The cell debris was removed by centrifugation at 18,000 g for 40 min at 4 °C. The supernatant was loaded on to an Ni–NTA column (GE Healthcare) equilibrated with 1 × PBS pH 7.0. The column was washed briefly with 1 × PBS pH 7.0 and then extensively with wash buffer (1 × PBS, 20 mM imidazole).The target protein was eluted with 1 × PBS containing 300 mM imidazole, then kept at 4 °C overnight in the presence of 0.3 mg ml^−1^ PPase to remove the tag. The protein was concentrated and purified using cation-exchange chromatography on a ResourceS column (GE Healthcare). The protein was further purified using a Superdex75 column (GE Healthcare) equilibrated with 1 × PBS. The purified protein was transferred into a buffer consisting of 20 mM Tris pH 8.0, 200 mM NaCl for crystallization. Selenomethionine (SeMet)-labelled YfiR protein was expressed using the methionine-auxotrophic *E. coli* strain B834 (DE3) (Novagen) in M9 minimal medium supplemented with 50mg selenomethionine per litre. The selenomethionine-labelled protein was purified using the same methods used for the wild-type YfiR protein.

The YfiR-YfiB(59–168) complex was prepared by expressing and purifying the two proteins separately, then mixing them together in a 1:1 molar ratio and concentrating to 10 mg ml^−1^. Formation of a stable complex was verified by SDS-PAGE analysis.

### GST pull-down

A similar amount of GST and GST-YfiR proteins were incubated with Glutathione Sepharose beads (GE Healthcare) (200 μl) at 4 °C for an hour with gentle agitation. The supernatant was then removed after centrifugation at 5,000 rpm for 4 min. The beads with GST proteins were washed four times with pre-cooled 1 × PBS, pH 7.0 buffer. Subsequently, equal amount of purified proteins [YfiB(59–168)WT, YfiB(59–168) M59R, YfiB(59–168) R96A] were incubated with the prepared beads at 4 °C for 3 h with gentle agitation. The beads were washed with 1 × PBSS, pH 7.0 buffer (1mL) four times. The supernatants were removed and the beads were resuspended with 1 × PBS (100 μl). Equal amounts of loading buffer were added, boiled and analyzed by SDS-PAGE. The protein bands were visualized by Coomassie Brilliant Blue R-250 (BioRad).

### Crystallization

The purified YfiB proteins were concentrated to 10 mg ml^−1^ and 20 mg ml^−1^. Initial crystallization screening by the sitting-drop vapour-diffusion method was performed at 293K using Crystal Screen kits (Hampton Research). Regular sheet-like crystals of YfiB(27–168) were observed after one week in a reservoir consisting of 0.1 M HEPES pH7.5, 2% PEG 400, 2.0 M ammonium sulfate. The crystals were briefly soaked in a cryoprotectant solution consisting of 3.5 M sodium formate and flash-cooled in liquid nitrogen prior to data collection. Regular club-shaped crystals of YfiB(59–168) were observed after one week from a reservoir consisting of 3.5 M sodium formate. The crystals were briefly soaked in a cryoprotectant solution consisting of 5 M sodium formate and flash-cooled in liquid nitrogen prior to data collection.

The purified mature YfiR protein was concentrated to 10 mg ml^−1^ and 20 mg ml^−1^. Initial crystallization screening by the sitting-drop vapour-diffusion method was performed at 293K using Crystal Screen kits (Hampton Research). Regular rhombic-shaped crystals were observed after one week from a reservoir consisting of 0.1 M HEPES pH7.5, 1.5 M lithium sulfate monohydrate. Crystals of selenomethionine-labelled protein were obtained from the same condition. The crystals were briefly soaked in a cryoprotectant solution consisting of 3.5 M sodium formate and flash-cooled in liquid nitrogen prior to data collection.

Initial crystallization screening for the YfiR-YfiB(59–168) complex was performed by the sitting-drop vapour-diffusion method at 293K using Crystal Screen kits (Hampton Research). Regular crystals of the complex were observed after one week from a reservoir consisting of 0.1 M HEPES pH 7.5, 1.8 M ammonium sulfate. The crystals were briefly soaked in a cryoprotectant solution consisting of 3.5 M sodium formate and flash-cooled in liquid nitrogen prior to data collection.

### Data collection

Prior to data collection, crystals were cryoprotected by adding 20% glycerol into the crystallization buffer (unless otherwise indicated) before being flash-cooled in liquid nitrogen. Diffraction data for native YfiR were collected on beamline 18U of the Shanghai Synchrotron Radiation Facility (SSRF) and data for Se-Met YfiR were collected on beamline BL-17A of the Photon Factory at 100K. Data were integrated, scaled and merged using the HKL2000 suite of programs^34^. Diffraction data for YfiB and YfiB-YfiR were collected on beamline 19U of the SSRF at 100K. Data were integrated, scaled and merged using the HKL3000 suite of programs^34^.

### Structure determination

The structure of *P. aeruginosa* YfiB(27–168) was determined by molecular replacement using the crystal structure of the peptidoglycan-associated outer membrane lipoprotein from *Yersinia pestis* CO92 (PDB ID: 4PWT, 79% coverage, 31% identity) as a search model using the Phaser^35^ module in PHENIX^36^. The structure was successfully built using the AutoBuild module in PHENIX, and refinement was performed in PHENIX with cycles of manual rebuilding in Coot^37^. The structure of *P. aeruginosa* YfiB(59–168) was determined by molecular replacement with Phaser using the YfiB(27–168) structure as a search model. Refinement was performed in PHENIX with cycles of manual rebuilding in Coot.

The structure of *P. aeruginosa* YfiR was determined by single-wavelength anomalous dispersion from a Se-Met derivative protein using the AutoSol module in PHENIX with 2.25 Å data collected at the peak wavelength. A total of 13 Se sites were located in two molecules with a figure of merit of 0.42. After an initial round of model building using the AutoBuild module in PHENIX, a total of 304 out of 310 residues were traced in two molecules with R_work_/R_free_ of 26.2%/29.6%. This model was then used for molecular replacement in PHENIX with a more complete 2.4 Å native data set. Refinement was performed in PHENIX with cycles of manual rebuilding in Coot.

The structure of *P. aeruginosa* YfiR-YfiB(59–168) was determined by molecular replacement in Phaser using the structures of YfiR and YfiB(59–168) as ensemble search models. The 1:1 complex was refined in PHENIX with cycles of manual rebuilding in Coot^37^. All structures were validated by MolProbity^38^.

### Analytical ultracentrifugation

Analytical ultracentrifugation (AUC) was performed using a using a Beckman Coulter XL-I analytical ultracentrifuge with two-channel centrepieces and sapphire windows at 42,000 rpm and 277K with interference detection. All proteins were assayed using a purified protein solution (400 μL, 3 mg ml^−1^) in loading buffer containing 20mM Tris-HCl, 200mM NaCl, with the exception of the YfiB-YfiR complex which was assayed at a concentration of 10 mg ml^−1^ in the same loading buffer. The data were analyzed using the SEDFIT software.

## Additional Information

**Accession codes:** Coordinates and structure factors have been deposited in the Protein Data Bank with the following accession codes: YfiR, PDB code 4ZHU; YfiB(27-168), PDB code 4ZHV; YfiB(59-168), PDB code 4ZHW; YfiR-YfiB(59-168), 4ZHY. 

**How to cite this article**: Li, S. *et al.* Structural insights into YfiR sequestering by YfiB in *Pseudomonas aeruginosa* PAO1. *Sci. Rep.* **5**, 16915; doi: 10.1038/srep16915 (2015).

## Supplementary Material

Supplementary Information

## Figures and Tables

**Table 1:** Data collection and refinement statistics.

	YfiB(27–168)	YfiB(59–168)	YfiR (Se-Met)	YfiR	YfiR(C71S)-YfiB(59–168)
Data collection statistics					
Space group	*P2*_*1*_ *2*_*1*_ *2*_*1*_	*P 4*_*1*_ *2*_*1*_ *2*	*P 4*_*3*_ *2*_*1*_ *2*	*P 4*_*3*_ *2*_*1*_ *2*	*C 2 2 2*_*1*_
Unit cell parameters (Å)	a = 36.8, b = 56.4, c = 139.6	a = b = 40.7, c = 155.2	a = 121.3, b = 121.3, c = 85.5	a = 121.3, b = 121.3, c = 85.5	a = 118.5, b = 161.0, c = 43.6
Resolution (Å)	50.0–1.58 (1.64–1.58)^a^	50.0–1.39 (1.41–1.39)	50.0–2.25 (2.33–2.25)	50.0–2.40 (2.48–2.40)	50.0–1.97 (2.00–1.97)
*R*_merge_ (%)^b^	6.9 (57.1)	13.6 (55.2)	8.8 (44.2)	9.4 (67.6)	11.2 (46.5)
*R*_meas_ (%)^c^	7.5 (62.0)	14.3 (60.1)	9.1 (46.9)	9.8 (71.0)	12.2 (52.3)
*I*/σ(*I*)	37.8 (1.9)	27.9 (1.5)	27.9 (2.3)	27.1 (2.8)	29.2 (2.0)
Completeness (%)	99.7 (100.0)	99.0 (95.6)	87.6 (58.8)	99.1 (99.6)	97.6 (80.2)
No. of observations	167,718	270,239	316,634	319,438	182,075
No. of unique observations	40,264 (2,000)	27,019 (1,264)	27,164 (1,782)	25,449 (2,344)	29,376 (1,178)
Redundancy	6.4 (6.4)	10.0 (8.5)	11.7 (8.0)	12.6 (10.0)	4.1 (4.0)
Refinement statistics					
Resolution (Å)	50.0–1.58	50–1.39		50.0–2.40	50.0–1.97
*R*_work_ /*R*_free_ (%)^d^	16.6/18.8	18.0/19.3		18.1/21.1	18.6/22.7
No. of non-hydrogen atoms					
macromolecules	2,107	843		2,290	2,007
water	277	242		109	160
Average B factor (Å^2^)					
macromolecule	33.8	20.2		45.6	48.7
solvent	40.7	34.4		45.3	47.5
R.m.s. deviations					
bond lengths (Å)	0.009	0.008		0.009	0.014
bond angles (°)	1.201	1.253		1.31	1.45
Ramachandran plot^e^					
Favoured (%)	98.1	100.0		97.3	98
Outliers (%)	0	0		0	0
MolProbity score^d^	1.23	1.20		1.49	1.74

^a^Numbers in parentheses are corresponding values for the highest resolution shell.

^b^*R*_*merge*_ = Σ_h_Σ_l_ | I_ih_− < I_h_ > |/Σ_h_Σ_I_ < I_h_ >, where < I_h_ > is the mean of the observations I_ih_ of reflection h.

^c^*R*_*meas*_ = {Σ_hkl_ [N/(N–1)]1/2 Σ_i_ | I_i_(hkl) – < I(hkl) > |}/Σ_hkl_ Σ_i_ I_i_(hkl), where I_i_(hkl) are the observed intensities, < I(hkl) > are the average intensities and N is the multiplicity of reflection hkl.

^d^*R*_*work*_ = Σ(||F_p_(obs)|−|F_p_(calc)||)/Σ|F_p_(obs)|; *R*_*free*_ = R factor for a selected subset (5%) of the reflections that was not included in prior refinement calculations.

^e^Ramachandran plot calculated using MolProbity^38^.
