# Supplementary material for: Structural insights into YfiR sequestering by YfiB in Pseudomonas aeruginosa PAO1
Source: Sci Rep. 2015 Nov 23;5:16915. doi: 10.1038/srep16915 (PMC4655355; doi:10.1038/srep16915)

Structural insights into YfiR sequestering by YfiB in *Pseudomonas aeruginosa* PAO1

Shanshan Li[1,2], Tingting Li[1,2], Yueyang Xu[2], Qionglin Zhang[2], Wei Zhang[2], Shiyou Che[1,2], Ruihua Liu[1,2], Yingying Wang[3]* & Mark Bartlam[1,2]*

Supplementary Information

Figure S1: The YfiB dimer interface. The YfiB dimer is shown in cartoon representation and coloured according to secondary structure using the scheme shown in Figure 1. Residues in the dimer interface are shown in stick representation. Inset: an enlarged view of the YfiB dimer interface. Residues in the dimer interface are shown in stick representation and labelled.

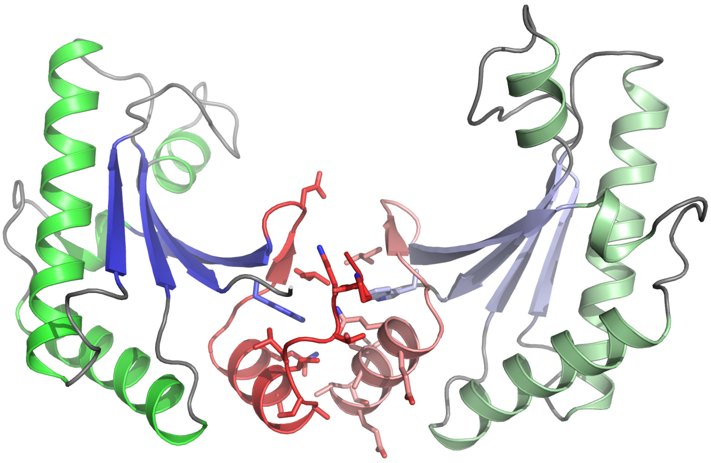

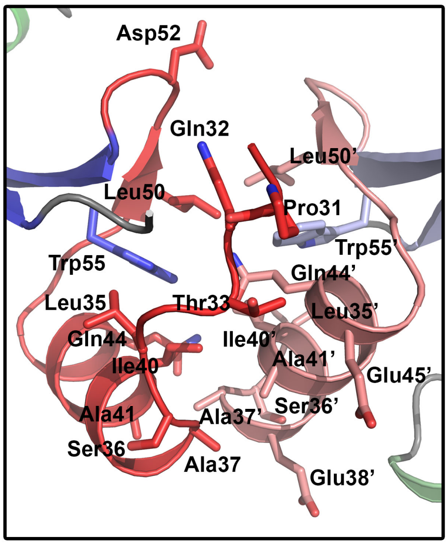

Figure S2: Analytical ultracentrifugation data for (A) L43P, (B) F48S, and (C) W55L mutants of YfiB.

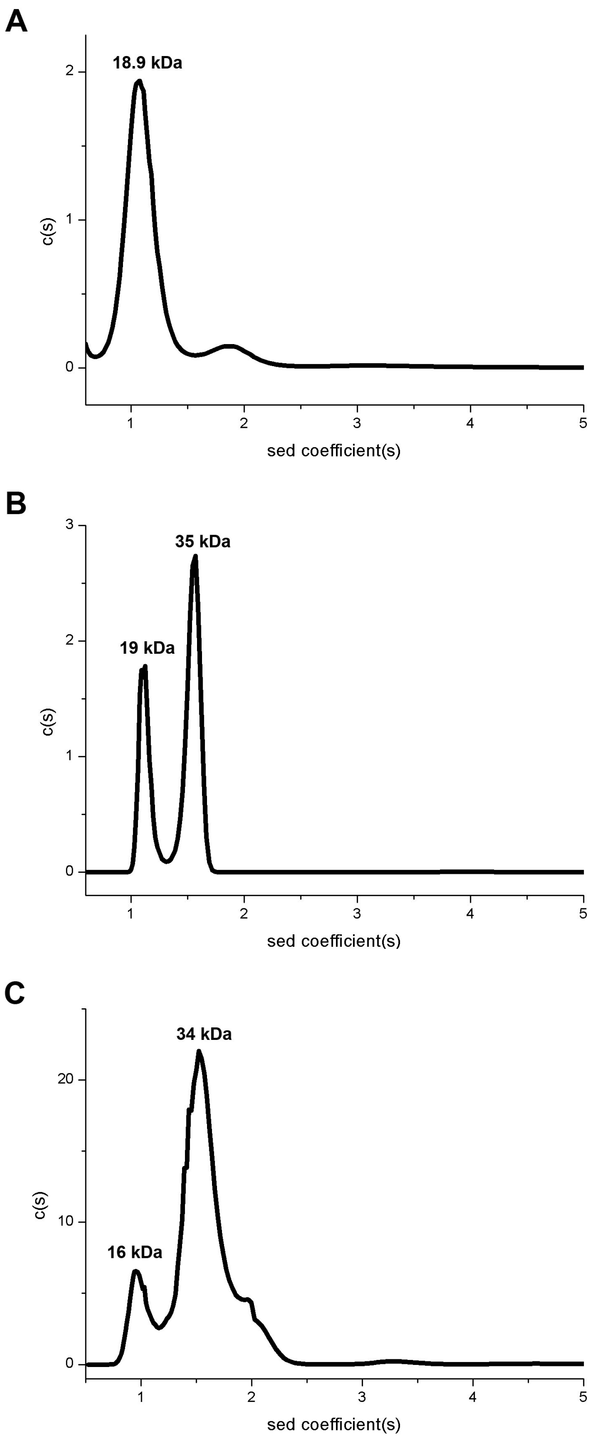

Figure S3: Multiple sequence alignment of YfiB. Sequences are shown for *P. aeruginosa*, *E. coli* and *K. pneumoniae* YfiB. The alignment is coloured according to % equivalency and secondary structure is shown for YfiB. The alignment was generated by Clustal Omega (http://www.ebi.ac.uk/Tools/msa/clustalo/) and displayed by JalView (http://www.jalview.org). Residues labelled with magenta asterisks are implicated in membrane attachment; residues labelled with cyan asterisks are implicated in PG binding.

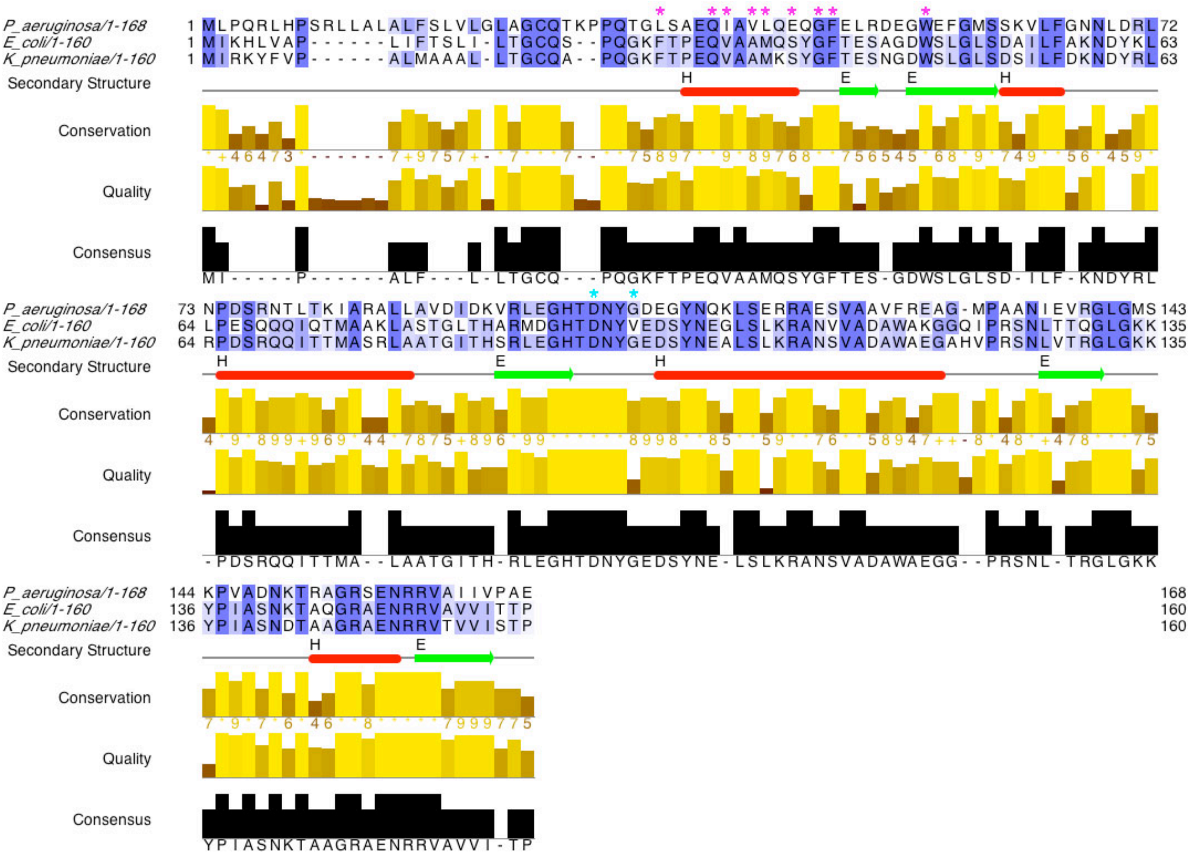

Figure S4: Analytical ultracentrifugation data for YfiR.

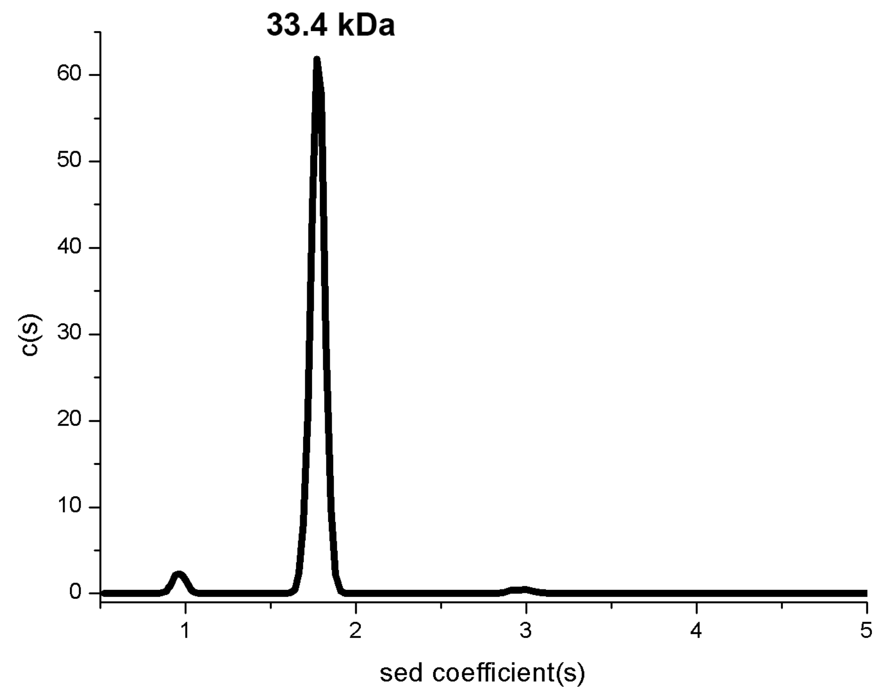

Figure S5: Multiple sequence alignment of YfiR. Sequences are shown for *P. aeruginosa*, *E. coli* and *K. pneumoniae* YfiR. The alignment is coloured according to % equivalency and secondary structure is shown for YfiR. The alignment was generated by Clustal Omega (http://www.ebi.ac.uk/Tools/msa/clustalo/) and displayed by JalView (http://www.jalview.org). Conserved cysteine residues are indicated by gold asterisks. Residues implicated in YfiN binding are indicated by magenta asterisks.

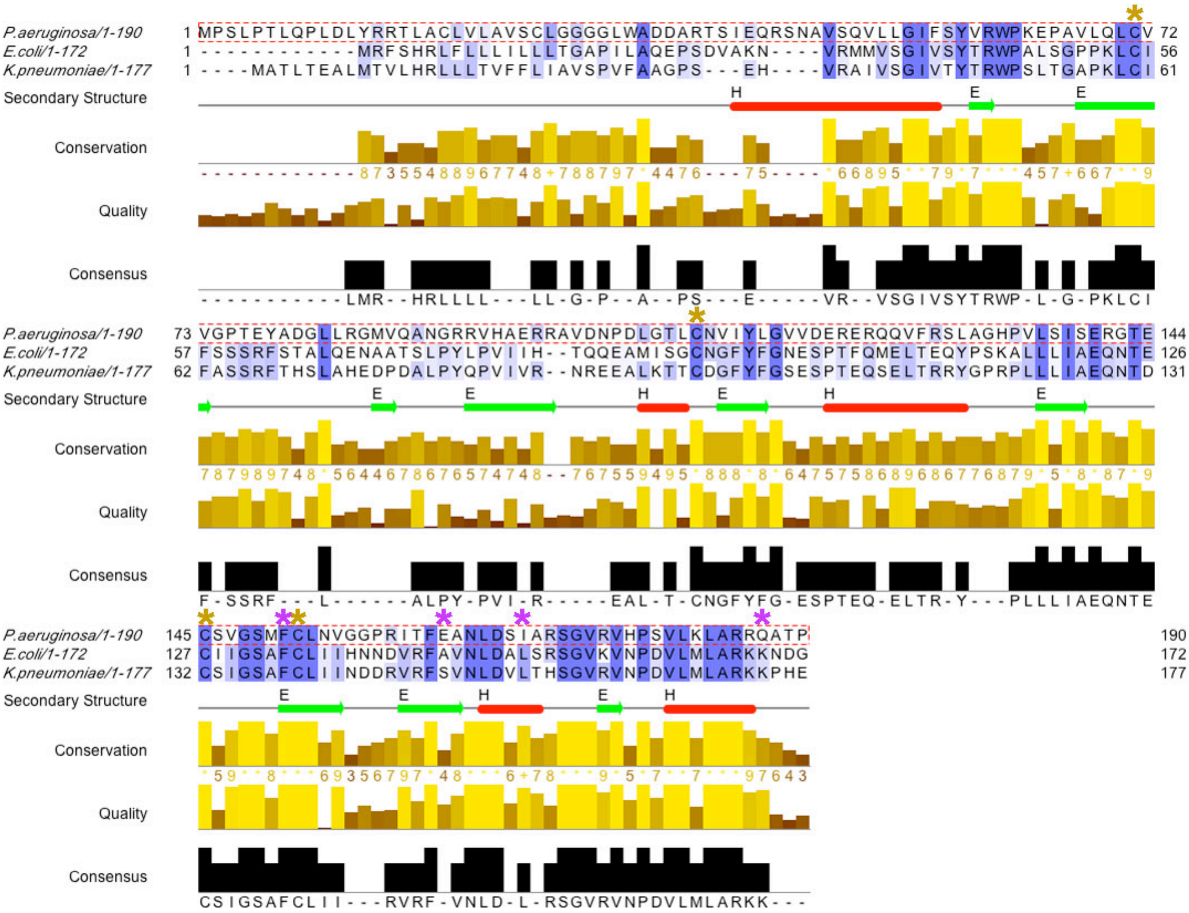

Supplement: Supplementary Information [file srep16915-s1.pdf]
